# Trigeminal Trophic Syndrome: A Case Report and Literature Review

**DOI:** 10.1055/a-2833-4794

**Published:** 2026-05-29

**Authors:** Han-Sang Song, Il-Hwan Kim, Hi-Jin You, Deok-Woo Kim, Tae-Yul Lee

**Affiliations:** 1 Department of Plastic and Reconstructive Surgery, Korea University Ansan Hospital, Ansan, Republic of Korea; 2 Department of Dermatology, Korea University Ansan Hospital, Ansan, Republic of Korea; 3 Institute of Advanced Regeneration and Reconstruction, College of Medicine, Korea University, Seoul, Republic of Korea

**Keywords:** trigeminal trophic syndrome, chronic ulcer, loss of nasal ala

## Abstract

Trigeminal trophic syndrome (TTS) is a rare neurocutaneous disorder defined by trigeminal anesthesia, facial paresthesia, and crescentic ulceration of the nasal ala. We describe an 86-year-old woman with long-standing trigeminal neuralgia who developed a suspicious ulcerated lesion on the left nasal ala. Although the initial biopsy showed atypical squamous cells suggestive of squamous cell carcinoma, the combined histopathologic, radiologic, and clinical findings supported a diagnosis of TTS. This case underscores the need to distinguish TTS from malignancy, particularly in elderly patients with neurologic comorbidities. Early recognition may prevent unnecessary invasive procedures and improve outcomes.

## Introduction

Trigeminal trophic syndrome (TTS) is an uncommon neurocutaneous disorder marked by trigeminal anesthesia, facial paresthesia, and a characteristically crescent-shaped ulcer of the nasal ala. Wallenberg first described TTS in 1901, and it usually follows trigeminal nerve injury; sensory loss and dysesthesias drive repetitive rubbing or picking, producing chronic ulceration within the trigeminal dermatome. 1 2 Diagnosis is often postponed because the lesion resembles disorders frequently encountered in plastic surgery, including squamous cell carcinoma, basal cell carcinoma, herpes infections, vasculitis, and pyoderma gangrenosum. 3

## Case

An 86-year-old woman was referred to our clinic to evaluate a left nasal-ala lesion suspected to be squamous cell carcinoma. Her history included hyperlipidemia, diabetes mellitus, hypertension, Parkinson's disease, and Alzheimer's disease. For trigeminal neuralgia, she had undergone trigeminal nerve blocks every 2 months for 8 years. The patient and her guardian provided written informed consent for photography.

**Fig. 1:**
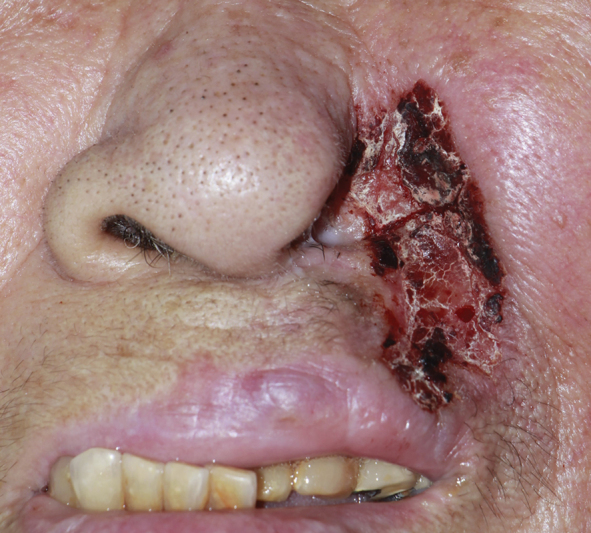
Clinical photograph of an erythematous, crusted patch on the nasal ala with subtotal tissue loss.

About 1 year before presentation, intense pruritus developed over the left nasal ala. She repeatedly rubbed and excoriated the area, and the wound gradually enlarged. She received oral antibiotics, topical corticosteroids, and topical antibiotics with gauze dressings for 1 year without benefit. After seeking care at several hospitals, she presented to our institution. Dermatologic examination showed a painful, erythematous, crusted patch with disruption of the nasal ala ( Fig. 1 ). A punch biopsy demonstrated atypical squamous cells, which raised suspicion for squamous cell carcinoma.

**Fig. 2:**
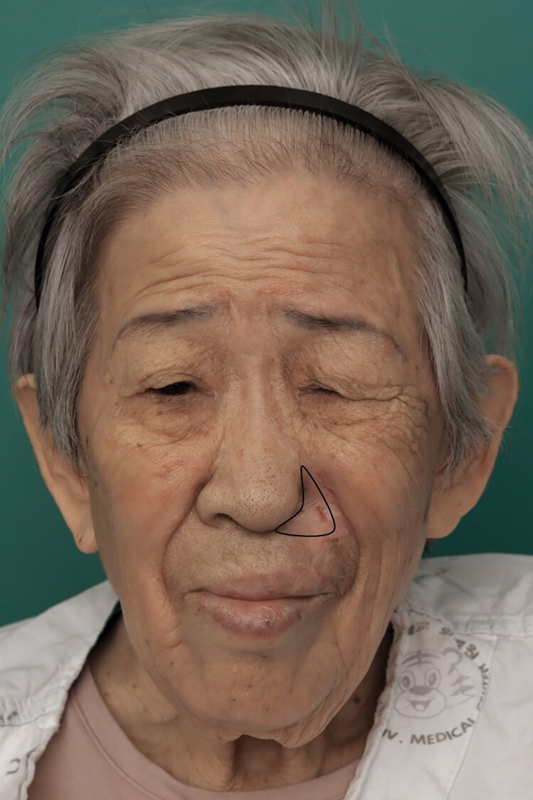
Clinical photograph showing improvement of the erythematous patch and residual sequelae after subtotal loss of the left nasal ala. The black line demarcates the area of subtotal ala loss.

She was referred for further management. Repeated nose rubbing with progressive loss of the nasal ala was unusual for squamous cell carcinoma, so we broadened the diagnostic evaluation. Facial magnetic resonance imaging showed no mass or abnormal enhancement of the left nasal ala or adjacent structures, and positron emission tomography–computed tomography revealed no abnormal uptake. A second biopsy obtained by our team showed chronic inflammation with fibrosis and no malignancy. In the setting of trigeminal neuralgia, negative imaging and pathology, and the characteristic ulcer pattern, we diagnosed TTS. Because of her age and comorbidities, we prioritized behavioral strategies to prevent further self-injury. Given her dementia, her caregiver monitored her closely and discouraged manipulation; she repeatedly reminded the patient not to touch the wound. We also applied protective dressings. The painful crusted patch improved, although the nasal ala remained disrupted ( Fig. 2 ).

## Discussion

TTS most often arises after trigeminal nerve damage, leading to sensory loss and dysesthesias. Iatrogenic injury predominates, particularly after ablative procedures for trigeminal neuralgia, such as rhizotomy or alcohol injection targeting the Gasserian ganglion. 4 Other reported causes include cortical and brainstem infarction, vertebrobasilar insufficiency, acoustic neuroma, astrocytoma, intracranial meningioma, spinal cord degeneration, *Mycobacterium leprae* neuritis, herpes zoster ophthalmicus, syringobulbia, postencephalitic Parkinsonism, and trauma. 4 Approximately 5% of cases are idiopathic. 1

Ulcers typically result from repeated mechanical injury to an area with diminished sensation. 5 Because patients feel little or no pain, scratching or pinching can continue even as ulceration progresses and tissue is lost. 6 Patients often describe paresthesias as burning, itching, or a crawling sensation. 7 Self-injurious behaviors are more common in individuals with psychiatric comorbidities, including anxiety disorders, obsessive–compulsive disorder, affective disorders, Alzheimer's disease, and factitious disorder. 8 Lesions most often affect the nasal ala, although the scalp, forehead, ear, palate, and jaw have also been reported. 9 The nasal tip is often spared because its innervation derives from the medial branch of the anterior ethmoidal nerve. 10 The mean age at presentation is 57 years, and the female-to-male ratio is 2.2:1. 11 The delay between nerve injury and ulceration ranges from weeks to decades. 11

**Fig. 3:**
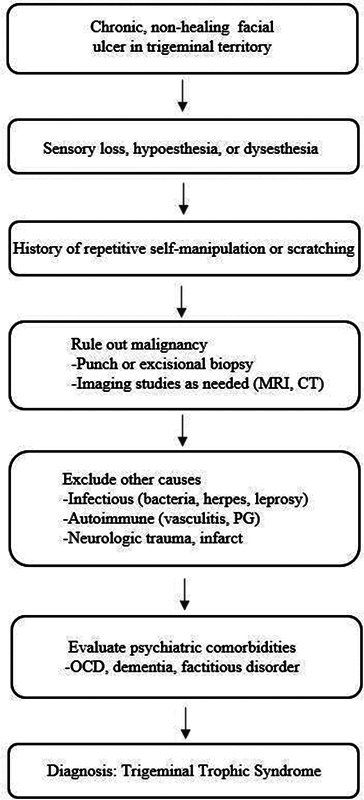
Diagnostic workflow for trigeminal trophic syndrome. OCD, obsessive–compulsive disorder; PG, pyoderma gangrenosum.

**Fig. 4:**
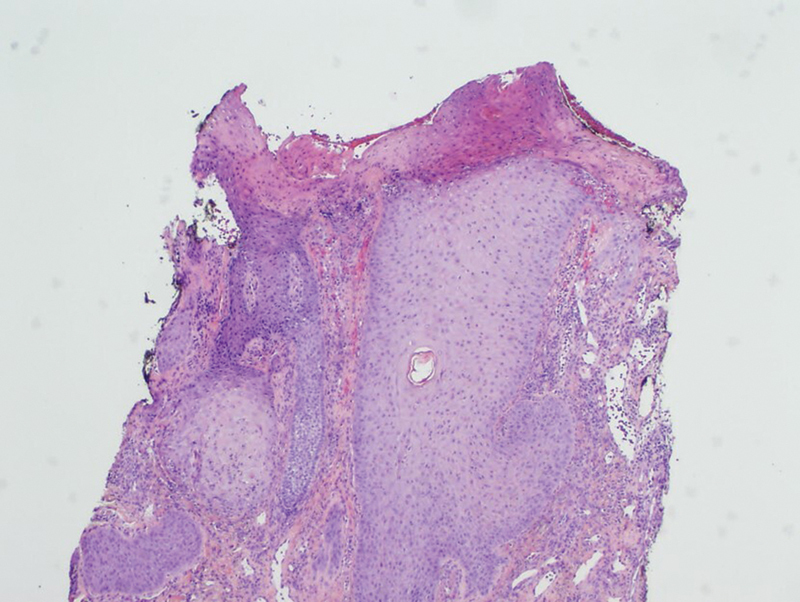
Epidermal hyperplasia encircles an acute ulcer with purulent scale-crust. The underlying granulation tissue contains patchy infiltrates composed of lymphocytes, histiocytes, and neutrophils (hematoxylin and eosin, ×40). Reproduced with permission from Khan AU, Khachemoune A. Trigeminal trophic syndrome: An updated review. *Int J Dermatol* 2019;58(5):530–537, with permission from John Wiley and Sons. 1

TTS is typically diagnosed only after other causes of facial ulceration are excluded ( Fig. 3 ). 1 The history should focus on prior trigeminal injury and on coexisting psychiatric disease. 12 Laboratory testing—complete blood count, basic chemistry, antinuclear antibody, rheumatoid factor, and cytoplasmic and perinuclear antineutrophil cytoplasmic antibody—is typically normal. 13 The differential diagnosis includes neoplasms, infections, systemic vasculitides, and pyoderma gangrenosum. 1 Histopathology is not specific, but a biopsy is required to rule out malignancy, infection, or vasculitis. 14 On hematoxylin and eosin staining, specimens usually show nonspecific inflammatory changes ( Fig. 4 ). 1 4 Clinically and histologically, TTS can resemble squamous cell carcinoma, especially when atypical squamous cells reflect reactive change from chronic inflammation. Atypical squamous cells can be caused by malignancy, premalignant change, or inflammation; inflammation is most common. Imaging supports exclusion of malignancy, and diagnosis may require repeated biopsies.

There is no universally accepted treatment, and management has relied on medications and surgery, as well as protective measures and transcutaneous nerve stimulation. 1 Education that the ulcer is self-inflicted is central to care. 15 Protective devices or prostheses, careful fingernail trimming, and cotton gloves can reduce additional trauma. 15 Agents reported to lessen paresthesias and compulsive behaviors include pimozide, carbamazepine, chlorpromazine, amitriptyline, diazepam, vitamin B supplements, and clonazepam. 4 Additional approaches include transcutaneous electrical stimulation, iontophoresis, nerve blockade, ionizing radiation, ipsilateral cervical sympathectomy, and stellate ganglionectomy. 4 Reconstructive surgery may succeed when patients can avoid further manipulation. 1 Reported techniques include a contralateral forehead flap, 13 which may disrupt the dysesthetic–trauma cycle and improve aesthetics, 16 as well as ipsilateral local cheek flaps and forearm free flaps. 1 Finucane et al reported three main strategies: Behavioral modification with dressings and protective devices, systemic pharmacotherapy, and surgery. Reported outcomes were 61.5% improvement and 26.9% resolution with behavioral measures; 63.1% improvement and 15.8% resolution with systemic pharmacotherapy; and 46.2% improvement and 34.6% resolution with surgery. For the forehead flap, outcomes were 41.7% improvement and 58.3% resolution ( Table 1 ). 17

**Table 1:** Treatment outcomes by therapeutic approach

	Improved ( *n* / *N* )	Improved (%)	Resolved ( *n* / *N* )	Resolved (%)
Behavioral modification with wound dressings	16/26	61.5%	7/26	26.9%
Systemic pharmacotherapy	24/38	63.1%	6/38	15.8%
Surgery	12/26	46.2%	9/26	34.6%
Forehead flap	5/12	41.7%	7/12	58.3%

TTS is an uncommon sequela of trigeminal nerve injury and is typically a diagnosis of exclusion. Clinicians should suspect it when a facial ulcer fails to heal, especially in older adults with a history of trigeminal neuralgia. Prompt recognition and conservative care often avert unnecessary operations and lead to better clinical outcomes. Plastic surgeons are well-positioned to identify TTS early, steer patients away from inappropriate procedures, and plan reconstruction when it is truly needed.
